# On optimal comparability editing with applications to molecular diagnostics

**DOI:** 10.1186/1471-2105-10-S1-S61

**Published:** 2009-01-30

**Authors:** Sebastian Böcker, Sebastian Briesemeister, Gunnar W Klau

**Affiliations:** 1 Institut für Informatik, Friedrich-Schiller-Universität, Jena, Germany; 2 Jena Centre for Bioinformatics, Jena, Germany; 3 Div. for Simulation of Biological Systems, ZBIT/WSI, Eberhard Karls Universität Tübingen, Germany; 4 CWI, P.O. Box 94079, 1090 GB Amsterdam, The Netherlands

## Abstract

**Background:**

The COMPARABILITY EDITING problem appears in the context of hierarchical disease classification based on noisy data. We are given a directed graph *G *representing hierarchical relationships between patient subgroups. The task is to identify the minimum number of edge insertions or deletions to transform *G *into a transitive graph, that is, if edges (*u*, *v*) and (*v*, *w*) are present then edge (*u*, *w*) must be present, too.

**Results:**

We present two new approaches for the problem based on fixed-parameter algorithmics and integer linear programming. In contrast to previously used heuristics, our approaches compute provably optimal solutions.

**Conclusion:**

Our computational results demonstrate that our exact algorithms are by far more efficient in practice than a previously used heuristic approach. In addition to the superior running time performance, our algorithms are capable of enumerating all optimal solutions, and naturally solve the weighted version of the problem.

## Background

Jacob *et al*. [1] raise the following problem from molecular diagnostics: For a group of patients that share a disease, we have to assign each patient to a well-defined subgroup in a hierarchical classification scheme of sub-diseases, based on molecular characteristics of the patient [2]. Such characteristics can be measured using high-throughput genomic approaches such as gene expression microarrays or SNP arrays, and may be accompanied by other data such as fluorescent in situ hybridization. The task is to deduce the hierarchical structure in the integrated noisy data sets by an automated approach. To do so, Jacob *et al*. [1] proceed in two steps: First, hierarchical dependencies between patient subgroups are stored in a directed graph, where vertices correspond to experimental characteristics (properties), and a directed edge between two properties *A *and *B *indicates that patients with property *A *are a (noisy) superset of patients with property *B*. Constructing this graph is straightforward using basic concepts from statistics: for example, one can choose a threshold *α *∈ [0, 1] and include an edge (*A*, *B*) if the ratio of patients with feature *B *that also exhibit feature *A *is greater or equal to *α *[1]. Parameter *α *can be used to scale the sparseness of the resulting graph.

The second step consists in *cleaning *the input graph: if property *A *is a superset for property *B*, and *B *is a superset for property *C*, then it is understood that *A *must also be a superset for property *C*.

Unfortunately, noise in the high-throughput data often leads to cases where edge (*A*, *C*) is not included during graph construction, even though edges (*A*, *B*) and (*B*, *C*) are present. From a graph-theoretical point of view, we require that for directed edges (*u*, *v*) and (*v*, *w*) present in the graph, also (*u*, *w*) has to be present. A graph that satisfies this property for all vertex triples is called *transitive*. If the input graph is not transitive, then it is natural to ask for the most parsimonious way to transform the input graph into a transitive graph, that is, the minimum number of edge modifications (insertions or deletions) such that the resulting graph is transitive. This problem is called *transitive approximation *[3] or COMPARABILITY EDITING [4], and we stick with the latter term. Important problem variants include WEIGHTED COMPARABILITY EDITING where each edge has individual modification costs, and enumerating *all *optimal solutions. Unfortunately, (weighted) COMPARABILITY EDITING is *NP*-complete [4]. Recently, Jacob *et al*. have presented a heuristic approach for the problem [1].

The COMPARABILITY EDITING problem is closely related to its undirected counterpart CLUSTER EDITING: Here, we are given an undirected graph as input, and our task is to find a set of edge modifications of minimum cardinality, such that the modified graph consists of disjoint cliques. This problem is also *NP*-complete [5]. To find exact solutions, we can formulate the problem as an integer linear program (ILP) and use a cutting plane approach for its solution [6]. Another way to find exact solutions for CLUSTER EDITING are fixed-parameter algorithms, using the number (or total cost) of edge modifications as parameter *k *[7,8]. The currently fastest algorithms for CLUSTER EDITING use both the ILP formulation and fixed-parameter algorithmics and, despite the hardness of the problem, allow to efficiently solve instances with thousands of vertices [9].

### Our contributions

We have adopted both the ILP formulation and the fixed-parameter algorithms (FPT) developed for CLUSTER EDITING to work with its directed counterpart, COMPARABILITY EDITING. Both approaches guarantee that *exact *solutions are found. In addition, FPT guarantees worst-case running times, with exponential dependency on the parameter *k *only. The ILP formulation is, in practice, several thousand-fold faster than the heuristics from [1] even for small instances with 25 vertices. Combining FPT data reduction and ILP to solve the remaining problem leads to best running times. Both FPT and ILP can enumerate all optimal solutions, and for FPT this is possible with almost no computational overhead. Both approaches also work for WEIGHTED COMPARABILITY EDITING, which might lead to better solutions, for example, in the molecular diagnostics application: one might use the difference to the threshold *α *as modification cost of an edge, so that edges with measurements close to the threshold can be inserted or deleted cheaper than edges that deviate by a large extent from the threshold.

## Methods

### Preliminaries

Throughout this paper, let *n *:= |*V*|. We sometimes assume that *V *= {1, ..., *n*}. We write *uv *as shorthand for an ordered pair (*u*, *v*) ∈ *V*^2^. For weighted instances, let *s *: *V*^2 ^→ ℝ encode the input graph: For *s*(*uv*) > 0 a directed edge *uv *is present in the graph and has deletion cost *s*(*uv*), while for *s*(*uv*) ≤ 0 the edge *uv *is absent from the graph and has insertion cost -*s*(*uv*). An unweighted COMPARABILITY EDITING instance can be encoded using *s*(*uv*) ∈ {± 1}. We call edges with *s*(*uv*) = ∞ *permanent *and with *s*(*uv*) = -∞ *forbidden*. Let *N*_+_(*v*) denote the *successors *of *v*, *N*_+_(*v*) = {*w *∈ *V *| *s*(*vw*) > 0}. Similarly, let *N*_-_(*v*) denote the *predecessors *of *v*, *N*_-_(*v*) = {*u *∈ *V *| *s*(*uv*) > 0}.

A directed graph *G *= (*V*, *E*) is *transitive *if for any three vertices *u*, *v*, *w *∈ *V *with *uv *∈ *E *and *vw *∈ *E *we also have *uw *∈ *E*. Any three vertices violating this condition are called a *conflict triple*.

### A fixed-parameter algorithm

The main idea behind many FPT graph modification algorithms is to localize and resolve forbidden substructures, either during preprocessing or in a search tree. In this paper, we transform any directed input graph *G *into a transitive graph by resolving all conflict triples. Our fixed-parameter algorithms sometimes require a maximum number of edge modifications *k *to be known in advance: To find an optimal solution we call this algorithm repeatedly, increasing *k*.

We first present methods for the data reduction of (unweighted and weighted) COMPARABILITY EDITING instances. We describe polynomial-time reduction rules and apply these rules over and over again until no further rule will apply.

#### Parameter-dependent data reduction

Our parameter-dependent data reduction is similar to that for CLUSTER EDITING in [8]. For every tuple *uv *we define induced costs *icf *(*uv*) and *icp*(*uv*) for marking *uv *as forbidden or permanent, respectively:

$$\begin{array}{lll} icf(uv) & = & \sum_{x\in N_{+}(u)\cap N_{-}(v)}\min\{s(ux),s(xv)\}\\ icp(uv) & = & \sum_{x\in N_{-}(u)\backslash N_{-}(v)}\min\{s(xu),-s(xv)\}\\ & & +\sum_{y\in N_{+}(v)\backslash N_{+}(u)}\min\{-s(uy),s(vy)\} \end{array}$$

We use these values and also take into account costs for inserting or deleting *uv*:

• For all *u*, *v *∈ *V *where

*icf*(*uv*) + max{0, *s*(*uv*)} > *k *:

Insert *uv *if necessary, and mark *uv *as permanent by assigning *s*(*uv*) ← +∞.

• For all *u*, *v *∈ *V *where

*icp*(*uv*) + max{0, -*s*(*uv*)} > *k *:

Delete *uv *if necessary, and mark *uv *as forbidden by assigning *s*(*uv*) ← -∞.

If there is a pair *uv *such that both conditions hold simultaneously, the problem instance is not solvable. To understand these rules, assume that an edge *uv *is *not *present in an optimal solution. Then, for edges *ux *and *xv *present in the input, either *ux *or *xv *(or potentially both) have to be deleted to make the graph transitive.

Clearly, we can also remove isolated vertices and edges. Unfortunately, we are currently not able to give a problem kernel for COMPARABILITY EDITING: A *problem kernel *is a set of reduction rules so that after exhaustive application of the rules, the remaining instance has size polynomial in the parameter *k *and independent of the original problem size *n *[10]. Our reduction rules are obviously not a problem kernel, because we never reduce the number of vertices in the graph except when vertices or edges become isolated. Constructing a problem kernel remains an interesting open problem.

If both *uv *and *vu *are permanent, we can *merge *vertices *u*, *v *into a new vertex *u*': For each vertex *w *∈ *V *\{*u*, *v*} we join *uw*, *vw *such that *s*(*u*'*w*) ← *s*(*uw*) + *s*(*vw*). Moreover, in case *w *is a non-common neighbor of *u*, *v *we can reduce *k *by min{|*s*(*uw*)|,|*s*(*vw*)|}. But it almost never happens in application that both *uv *and *vu *are permanent simultaneously, so this technique can rarely be applied. In particular, if the input graph is acyclic then no vertices will ever be merged.

#### Algorithm engineering and parameter-independent data reduction

First, we describe a fast method to compute a lower bound on the cost of WEIGHTED COMPARABILITY EDITING: Let *CT *be a set of edge-disjoint conflict triples. Then,

$$\sum_{uvw\in CT}\min\{s(uv),s(vw),-s(uw)\}$$

is a lower bound for solving all conflict triples in *CT *. Since finding the set *CT *maximizing this value is computationally expensive, we greedily construct a set of edge-disjoint conflict triples *CT *and use the above sum as a lower bound. We can use such lower bounds to make induced costs *icf *(*uv*) and *icp*(*uv*) tighter: let *b*(*G*, *uv*) be a lower bound that ignores all edges *uw*, *wu*, *vw*, and *wv *in its computation, for all *w *∈ *V *\{*u*, *v*}. Then, we can set an edge to forbidden or permanent if

icp_*_(*uv*) := *icp*(*uv*) + max{0, -*s*(*uv*)} + *b*(*G*, *uv*) > *k*

or

icf_*_(*uv*) := *icf*(*uv*) + max{0, *s*(*uv*)} + *b*(*G*, *uv*) > *k*

holds, respectively.

We now utilize an idea from [9] to transform the above parameter-dependent data reduction into a parameter-independent one. Therefore we use an *upper bound *for the modification costs of *G *as our parameter *k*. Without the knowledge of a current parameter both parameter-dependent data reduction rules can now be applied during the preprocessing. To calculate the upper bound we use a greedy approach that iteratively searches for edges where reduction rules almost apply. In detail, we search for an edge *uv *such that max{icp_*_(*uv*), icf_*_(*uv*)} is maximum and set *uv *to forbidden or permanent, respectively. Note that we can use any other upper bound for this reduction as well. The combination of lower and upper bounds makes this reduction very effective in application.

In our preprocessing, we mark an edge as permanent or forbidden when we can guarantee that this edge is always or never part of an optimal solution, respectively. This will usually not reduce the size of the instance. However, we will see in the section devoted to our computational results that in practice, adding information about permanent and forbidden edges makes it easier to solve the remaining instance. Note that we can process the reduced instance with *any *exact method or even heuristics.

#### Branching strategy

After parameter-independent data reduction, the remaining instance can be solved using a branching tree strategy. In such algorithms, we identify a conflict triple and then branch into sub-cases to repair this conflict. Let *uvw *be a conflict triple so that *uv *and *vw *are edges but *uw *is not. Recursively branch into three cases:

1. Insert *uw*, mark *uv*, *uw*, and *vw *as permanent.

2. Delete *uv*, mark *vw *as permanent, and *uv *and *uw *as forbidden.

3. Delete *vw*, mark *vw *as forbidden.

In each branch, we lower *k *by the insertion or deletion cost required for the executed operation. When a connected component decomposes into two components, we calculate the optimum solutions for these components separately. If *k *falls below zero, we discard the respective branch of the algorithm. Keeping in mind that we can find a conflict triple in time *O*(*n*^3^), the branching algorithm has running time *O*(3^*k*^·*n*^3^). Unfortunately, we cannot replace the polynomial factor by a summand because no polynomial-size problem kernel is known for the problem.

As an algorithm engineering technique, we do not process conflict triples in an arbitrary order but instead, choose that conflict triple *uvw *such that *icp*(*uv*) + *icp*(*vw*) + *icf*(*uw*) is maximal. Doing so, we choose a triple that results in a comparatively small branching number while avoiding the time-consuming exact computation of branching numbers. The branching number is the root of the characteristic polynomial of the branching vector and governs the asymptotic size of the search tree, see again [10] for details. To find an optimal solution we call the algorithm repeatedly, increasing *k *in an interval defined by lower and upper bound for this problem instance. While traversing the search tree, we apply reduction rules in every recursion step. Clearly, lower bounds as described in the section on parameter-independent data reduction, can also be used to stop search tree recursion more efficiently.

It is understood that our algorithm can also enumerate all optimal solutions, by completely traversing the search tree.

### An integer linear programming approach

Let *x *be a binary decision vector with *x*_*ij *_= 1 if directed edge (*i*, *j*) is part of the solution and *x*_*ij *_= 0 otherwise. Then, an optimal solution to WEIGHTED COMPARABILITY EDITING can be found by solving

(1) $$\min\sum_{ij\in E}s(ij)-\sum_{i=1}^{n}\sum_{j=1}^{n}s(ij)x_{ij}$$

(2) s. t. *x*_*ij *_+ *x*_*jk *_- *x*_*ik *_≤ 1   ∀1 ≤ *i*, *j*, *k *≤ *n*

(3) *x*_*ij *_= 0   ∀*ij *with *s*(*ij*) = -∞

(4) *x*_*ij *_= 1   ∀*ij *with *s*(*ij*) = ∞

(5) *x*_*ij *_∈ {0, 1}   ∀1 ≤ *i*, *j *≤ *n*.

**Figure 1:**
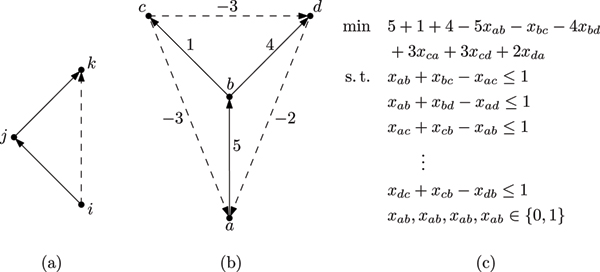
**The integer linear programming approach**. (a) Conflict triple, (b) small example instance of WEIGHTED COMPARABILITY EDITING, and (c) corresponding integer linear program. Solid edges are present in the graphs, dashed edges are absent. Edges missing from the complete directed graph in (b) have zero weight.

The *n*^3 ^*triangle inequalities *(2) of the ILP ensure that no conflict triple as shown in Fig. 1(a) occurs in the solution and model exactly the definition of transitivity. Equations (3) and (4) exclude forbidden edges and force permanent edges to be part of the solution.

Let $\bar{x}$ be a feasible solution of the ILP and let $\bar{E}$ be the corresponding edge set. It is easy to see that the graph *G *= (*V*, $\bar{E}$) is transitive. The objective function properly sums up the deletion and insertion costs as detailed by Table 1.

**Table 1:** Deletion and insertion costs used in the objective function of the ILP approach.

	*ij *∈ $\bar{E}$	*ij *∉ $\bar{E}$
(*i*, *j*) ∈ *E*	*s*(*ij*) - *s*(*ij*) = 0	*s*(*ij*) - 0 > 0
(*i*, *j*) ∉ *E*	-*s*(*ij*) > 0	0

Thus, an optimal solution of the ILP corresponds to an optimal solution of WEIGHTED COMPARABILITY EDITING. Figures 1(b) and 1(c) show a small example instance of the problem and the corresponding ILP. The ILP formulation basically describes the partial order polytope of a complete directed graph.

Müller [11] has investigated the facial structure of partial order polytopes and his results on facet-defining inequalities and their separation can directly be used to develop an effective branch-and-cut algorithm for WEIGHTED COMPARABILITY EDITING:

We start optimizing the LP relaxation (1) with an empty constraint set. Let $\bar{x}$ denote the vector corresponding to an intermediate solution of the linear programming relaxation. We first check whether $\bar{x}$ violates any triangle inequalities. If this is the case, we add the violated inequalities, resolve, and iterate. Otherwise, we check whether $\bar{x}$ is integral. If so, we stop, and $\bar{x}$ is an optimal solution. If, however, $\bar{x}$ has fractional entries, we may separate additional facet-defining inequalities. Müller proposes to focus on *odd closed walk inequalities *and presents an efficient separation algorithm in [11]. If we find cutting planes in the separation procedure we iterate, otherwise we branch. Applying the cutting plane method at each node of the branch-and-bound tree leads to a *branch-and-cut algorithm*.

Note that in our current implementation we have not yet realized additional cutting planes because separating triangle inequalities proved to be sufficient for the tested instance sizes. Once larger or more complicated instances have to be dealt with, the separation of odd closed walk inequalities seems to be a promising direction to approach new orders of magnitude in instance size.

To enumerate solutions with the ILP approach, we propose the following, straightforward iterative cutting plane approach. Cutting of a given solution x*, as done in line 3 of the algorithm, can be realized by adding inequality

(6) $$\sum_{i|x_{i}^{*}=0}x_{i}+\sum_{i|x_{i}^{*}=1}(1-x_{i})\geq 0$$

1 find first optimal solution x* with value *z*_0 _= z*;

2 **repeat**

3   add cutting plane (6) which cuts off x*;

4   find next optimal solution x* with value z*;

5 **until **z* > *z*_0 _*or ILP infeasible *;

## Results and discussion

We evaluate the performance of our algorithms on three datasets. First, Jacob *et al*. [1] synthetically created graphs for a fixed number of nodes *n *and an edge probability *p*. Two vertices *u*, *v *∈ *V *:= {1, ..., *n*} with *u *<*v *are connected by a directed edge (*u*, *v*) with probability *p*. For each combination of *n *∈ {10, 15, 20, 25} and *p *∈ {0.1, 0.2, ..., 0.9} they generated 20 graphs. On average, these graphs are most distant from the transitive state for *p *= 0.5.

Second, we generated a dataset of larger graphs. We defined these graphs by the number of nodes *n *and the number of edge changes *k*. Initially, we generate a transitive graph that has *n *nodes *V *:= {1, ..., *n*} and contains all $\left(\begin{array}{c} n\\ 2 \end{array}\right)$ directed edges of the form (*u*, *v*) for *u *<*v*. Next, we choose *k *distinct vertex tuples *uv *∈ *V*^2 ^and delete or insert the corresponding edges. The resulting graph has distance at most *k *to a transitive graph.

Third, we also evaluate our algorithms on the biological dataset from [1], which results from an extensive study with patients suffering from mature aggressive lymphomas. Here, we demonstrate that the FPT approach can enumerate all optimal instances in short computation time.

### Performance of reduction rules

Combining FPT reduction rules with upper and lower bounds allows us to reduce input graphs in advance. In contrast to the CLUSTER EDITING problem this will usually not reduce the graph size. However, we can mark some edges in the graph as permanent or forbidden. We measure the reduction ratio by estimating the real modification costs using the mean of upper and lower bound. The *reduction ratio *is defined as $1-\frac{costs_{\text{reduced}}}{costs_{\text{original}}}$, where *costs*_original _and *costs*_reduced _are the estimated modification costs before and after the reduction. Table 2 shows that reduction of the first artificial dataset is less effective for edge probabilities around *p *≈ 0.5. We attribute this to the fact that graphs with *p *≈ 0.5 are almost random and have strong local defects, whereas other graphs are closer to the transitive state. Average running times for the reduction are between 4 ms for graphs with 10 vertices, and 95 ms for 25 vertices.

**Table 2:** Reduction ratios for artificial data by Jacob et al. Average reduction ratio for different edge probabilities. Each group contains 80 instances.

edge probability	0.1	0.2	0.3	0.4	0.5	0.6	0.7	0.8	0.9
avg. reduction ratio	0.33	0.20	0.08	0.05	0.03	0.15	0.24	0.21	0.74

Our data reduction reduces graphs even more effectively if they are large and close to the transitive state. Using the second artificial dataset, we found that if the ratio *k*/*n *is less than 3, our reduction achieves a reduction ratio of 98.5%. This is in fact very promising for biological data since here, input graphs are usually close to the transitive state. Average running times for the reduction are between 4.1 s for graphs with 100 vertices, and 5.9 min for large graphs with 500 vertices.

### Artificial data by Jacob et al

**Figure 2:**
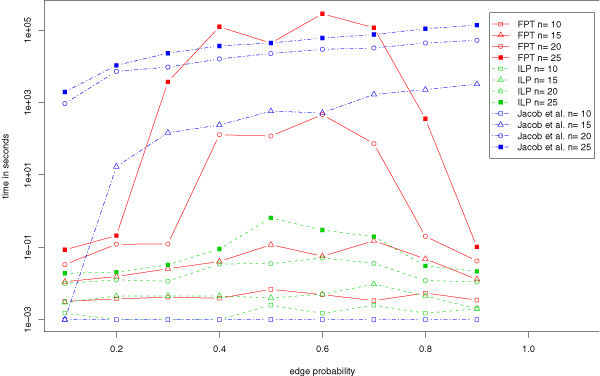
**Artificial data by Jacob et al**. Performance of the heuristic approach of Jacob *et al.*, the FPT algorithm, and the ILP approach.

We compare the running times of our exact FPT and ILP algorithms to the running times of the heuristic algorithm of Jacob *et al*. [1]. The reader should keep in mind that both our algorithms *guarantee *to find optimal solutions. Figure 2 shows running times for different graph sizes. Clearly, the performance of the algorithms differs significantly. Running times of the heuristic approach continually increase with greater edge probability. In contrast, our FPT and ILP algorithms show highest running times for the most "complicated" graphs with edge probability around 0.5. Clearly, running times of all three algorithms increase for larger graph size. The ILP algorithm outperforms the other two algorithms for graphs with more than 10 nodes by several orders of magnitude and computes provably optimal solutions for all instances in less than a second. The FPT algorithm shows good performance for nearly transitive graphs and solves them in less than an hour. Table 3 shows some detailed running times.

**Table 3:** Runtimes for artificial data by Jacob et al. Average runtimes of the heuristic approach of Jacob et al. [1], the FPT algorithm, and the ILP approach. (*) The FPT algorithm was not able to solve 17 instances of size 25 with edge probability 0.5 within 7 days of computation.

size	10			25		
edge probability	0.2	0.5	0.8	0.2	0.5	0.8
avg. cost	2.35	6.15	5.95	20.2	60.5	46.6
time Jacob *et al*.	< 0.2 s	< 0.2 s	< 0.2 s	2.95 h	12.2 h	30.4 h
time FPT	4 ms	7 ms	5 ms	209 ms	12.2 h*	5.9 min
time ILP	< 0.5 ms	1.5 ms	0.5 ms	2 ms	0.65 s	30 ms

**Figure 3:**
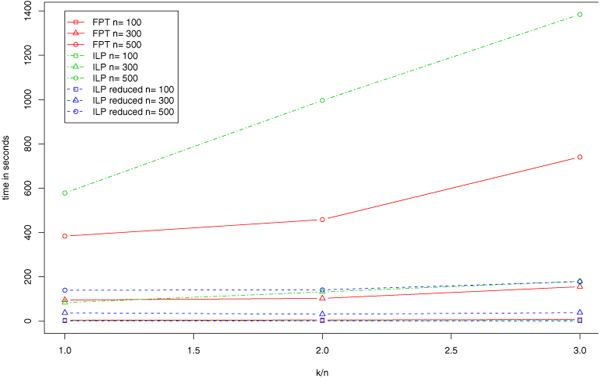
**Larger artificial data**. Average runtimes of the FPT algorithm, the ILP approach, and the ILP approach on preprocessed instances.

Most graphs derived from real-world data are almost transitive. This is due to the fact that the "true" graph is known to be transitive but the input graph suffers from some noise in the data. Our second benchmark set of larger artificial graphs accommodates this aspect, and we compare FPT and ILP on this dataset. To allow for a fair comparison, we first apply our data reduction preprocessing, and solve the reduced instances with FPT branching and the ILP algorithm. Figure 3 shows the running times of all three approaches. All approaches can solve large instances with up to 1 500 edge modifications in a matter of minutes. Again, performance of the ILP approach strongly depends on the graph size and less pronounced on the number of modifications. The opposite can be observed for the FPT algorithm. Since the benchmark set contains large graphs with moderate modification costs, the FPT algorithm outperforms the ILP algorithm on this data. However, in case we use our data reduction in advance, running time of the combined FPT-ILP approach is significantly smaller than those of both algorithms and shows the best overall performance. Our results indicate that both algorithms are suitable for even larger graphs.

### Lymphoma dataset

**Figure 4:**
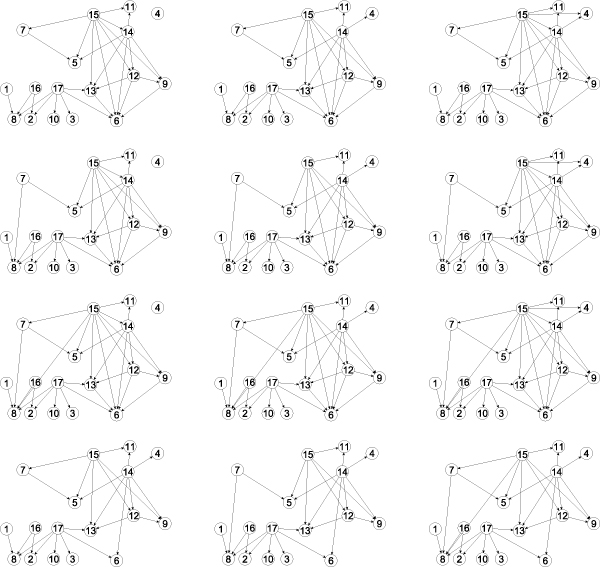
**Lymphoma dataset from **[1]** (mature aggressive B-cell lymphoma)**. All twelve optimal solutions enumerated in 25 ms with the FPT approach. The node numbers correspond to the molecular characteristics given in Table 4.

Finally, we run the FPT algorithm on the data on mature aggressive B-cell lymphoma from Jacob *et al*. and enumerate all optimal solutions. This results in twelve optimal solutions shown in Fig. 4. All optimal solutions were enumerated in only 25 ms. In contrast, Jacob *et al*. [1] report only six optimal solutions that were found by their heuristic.

**Table 4:** Lymphoma dataset from [1] (mature aggressive B-cell lymphoma). Mapping of node numbers in Figure 4, the abbreviations used in [1], and the molecular characteristics.

node nr	abbrev. in Jacob *et al*.	molecular characteristic
1	CD10	Antibodies for biomarker CD10 are present in histological section of lymphoma tissue
2	Ki-67	Antibodies for biomarker Ki-67 are present in histological section of lymphoma tissue
3	CD5	Antibodies for biomarker CD5 are present in histological section of lymphoma tissue
4	bcl6BR	Breakpoint in the BCL6 locus
5	IGH-BCL2	Fusion of BCL locus to immunoglobulin IGH
6	ABC	Gene expression profile similar to activated B-cells
7	GCB	Gene expression profile similar to germinal center B-cells
8	mBL	Gene expression signature which characterices molecular Burkitt lymphoma (Hummel *et al*.)
9	non-mBL	Absent gene expression signature non-mBL.
10	IG-MYC	Translocation of MYC locus involving fusion of MYC to immunoglobulins IGH, IGK or IGL
11	atyp.myc	Breakpoint in the MYC locus without fusion to an immunoglobulin
12	MYC-	No abberation of the MYC locus
13	CD10-	Antibodies for biomarker CD10 are absent in histological section of lymphoma tissue
14	Ki-67-	Antibodies for biomarker Ki-67 are absent in histological section of lymphoma tissue
15	CD5-	Antibodies for biomarker CD5 are absent in histological section of lymphoma tissue
16	bcl6BR-	No breakpoint in the BCL6 locus
17	IGH-BCL2-	No fusion of BCL locus to immunoglobulin IGH

## Conclusions and outlook

We have studied the WEIGHTED COMPARABILITY EDITING problem and have presented two exact algorithms for its solution. Our experimental results demonstrate that the exact approaches significantly outperform the heuristic approach proposed in [1]. In addition to the superior running time performance, our algorithms are capable of enumerating all optimal solutions, and naturally solve the weighted version of the problem.

In the future we plan to implement the full cutting plane approach including the odd closed walk inequalities proposed by Müller [11] and expect an even better performance behavior of the ILP approach. On the FPT side, constructing a problem kernel remains an interesting open problem. Further, we want to study the weighted problem variant more intensively, since weighted directed graphs seem to be more realistic models of molecular properties than unweighted ones. Labeling edges of molecular graphs with probabilities or log-likelihoods may lead to fewer and medically more meaningful optimal solutions and may also help to distinguish good solutions from false positive transformations. Finally, a more precise ranking of multiple (optimal and non-optimal) solutions might prove beneficial for the interpretation of results, and also hints on the "reliability" of edges. Clearly, detecting hierarchical relationships in noisy data may have applications that go beyond hierarchical disease classification.

The source code of our reduction and comparability editing tools, as well as the data used in this article is publicly available as the charles package of the open software library planet-lisa [12]. Furthermore, we plan to implement a web interface for our tools in order to give a large community access to our exact clustering and comparability editing tools and to facilitate comparison and evaluation.

## Competing interests

The authors declare that they have no competing interests.

## Authors' contributions

SBö, SBr, and GWK jointly conducted the research, performed the experiments, and wrote the paper.
